# Classification of fashion e-commerce products using ResNet-BERT multi-modal deep learning and transfer learning optimization

**DOI:** 10.1371/journal.pone.0324621

**Published:** 2025-05-22

**Authors:** In-Jae Seo, Yo-Han Lee, Beakcheol Jang

**Affiliations:** Graduate School of Information, Yonsei University, Seoul, Seodaemun-gu, Republic of Korea; Hebei University of Technology, CHINA

## Abstract

As the fashion e-commerce markets rapidly develop, tens of thousands of products are registered daily on e-commerce platforms. Individual sellers register products after setting up a product category directly on a fashion e-commerce platform. However, many sellers fail to find a suitable category and mistakenly register their products under incorrect ones. Precise category matching is important for increasing sales through search optimization and accurate product exposure. However, manually correcting registered categories is time-consuming and costly for platform managers. To resolve this problem, this study proposes a methodology for fashion e-commerce product classification based on multi-modal deep learning and transfer learning. Through the proposed methodology, three challenges in classifying fashion e-commerce products are addressed. First, the issue of extremely biased e-commerce data is addressed through under-sampling. Second, multi-modal deep learning enables the model to simultaneously use input data in different formats, which helps mitigate the impact of noisy and low-quality e-commerce data by providing richer information.Finally, the high computational cost and long training times involved in training deep learning models with both image and text data are mitigated by leveraging transfer learning. In this study, three strategies for transfer learning to fine-tune the image and text modules are presented. In addition, five methods for fusing feature vectors extracted from a single modal into one and six strategies for fine-tuning multi-modal models are presented, featuring a total of 14 strategies. The study shows that multi-modal models outperform unimodal models based solely on text or image. It also suggests the optimal conditions for classifying e-commerce products, helping fashion e-commerce practitioners construct models tailored to their respective business environments more efficiently.

## Introduction

The e-commerce market is developing rapidly due to the advancement of online and communication technology, as well as the convenience and speed of shopping using smartphones. Korea’s e-commerce transactions, including mobile and PC shopping, in October 2024 amounted to KRW 20.2845 trillion [1]. Naver Shopping (https://shopping.naver.com/), Korea’s largest e-commerce platform, matches more than 20 million newly registered products to approximately 5,000 categories every day so that customers can easily search for the products they need and systematically manage them [2]. Other shopping platforms such as ZigZag (https://zigzag.kr/), Musinsa(https://musinsa.com/) or Ably(https://m.a-bly.com/) reached more than KRW 100 billion sales in 2024 [3]. However, it takes considerable time and money to manually match a large number of products registered in real time to appropriate categories. Sellers using e-commerce platforms have difficulty registering products by selecting the appropriate categories for the products they sell. If a product is registered in the wrong category, it is not exposed to the search results of the customers who purchase it, and the sales rate of the product decreases. Thus, category classification plays an important role for platform operators, sellers, and consumers. However, it is nearly impossible to manually manage numerous products. If category classification is viewed as a typical classification problem of deep learning and is automated using deep learning, it can be appropriate for problem solving, as it can save time and money required for category matching work. However, product category classification using deep learning is difficult for three reasons. First, the number of products registered in each category is extremely biased owing to the characteristics of e-commerce data. Second, product information data such as product photos, titles, and categories are low-quality data in which incorrect or unimportant information is often entered in the process of direct preparation by sellers. Third, it takes considerable computing resources and learning time to train a deep learning model using different types of data, such as the images, text, and numeric data generated when many products are registered.

Imbalanced data have a significant impact on accuracy of classification problems [4]. One method used to solve the data imbalance problem is under-sampling. This method solves data imbalance by deleting some data from a class with many data compared to other classes. Anand et al. [5] describe the under-sampling technique solving the imbalance problem of biological data and improved the performance of class classification. In this study, the data imbalance problem was solved by using an under-sampling technique to extract the same amount of data from each class. Next, multimodal deep learning can be used to provide more information by using various types of data simultaneously for accurate category classification of low-quality data. Research on classifying product categories using only product images was conducted in [6,7]. However, there may be images that do not have high-quality visual information that is representative of the category. In the case of low-quality image data, the feature information for image classification cannot be sufficiently extracted, and the classification accuracy may decrease. To overcome these limitations, there is a multi-modal classification method that simultaneously utilizes product title data in the form of text along with product images. If information is simultaneously extracted from modalities that have different information and used as information for category classification, the accuracy of the model can be increased. Finally, it is possible to reduce the learning time and use of computing resources by utilizing transfer learning [8,9], which uses a neural network that has been trained with a large amount of data in advance. Transfer learning is effective and learns quickly, even when the number of data is small. In addition, higher accuracy can be obtained compared to the case of learning from scratch without transfer learning. In this study, we applied the above three methods to the image data and product title text data of fashion products, which is a representative industry of the e-commerce business, to classify their categories. While solving the imbalance problem of category data, the number of data was reduced through under-sampling, in which data with an extremely small number of products in category were excluded from model training. To compensate for this, transfer learning was used to learn quickly while reducing the performance loss, even with relatively small amounts of data. Finally, by using image and text data simultaneously, each feature was extracted, and categories were classified using more information than when using only individual data. In addition, to identify transfer learning and multimodal modeling methods optimized for the fashion category, various modeling methods were tested and models that showed optimal results were identified. If product categories can be quickly and accurately classified using the methodology proposed in this study, the products registered on the e-commerce platform can be systematically managed and the platform operator can efficiently operate the platform. Consumers can accurately and quickly search for products and thus find and purchase the products they need. Sellers can increase their sales if their products are exposed by accurately responding to consumers’ searches. In other words, the multimodal deep learning category classification using transfer learning proposed in this study can provide helpful value to sellers, consumers, and platform operators in platform business [10].

## Related works

### Image-based classification

Convolutional neural networks (CNNs) are widely used in the field of visual recognition and exhibit good performance. CNNs are actively used in basic visual recognition tasks such as image classification, object detection, semantic segmentation, and object localization [10,11]. After AlexNet [12] showed good results in the field of image classification, many researchers started researching the improvement of performance by constructing a deep convolutional neural network (DCNN) architecture that deeply stacks CNNs. The Visual Geometry Group (VGG) [13] and GoogLeNet [14] have demonstrated that combining deep and wide CNN layers improves performance. However, simply stacking layers deeply causes problems with the vanishing/exploding gradients. In addition, deep networks can fail to converge to the performance optimization point and cause degradation problems [11]. ResNet was proposed by He et al. [11] to solve the problems that arise as the network deepens. These problems were solved through identity mapping, which adds the input value as it is to the output value that passed through one learned layer in the residual learning block, rather than simply stacking the layers deeply. Recently, Macfadyen et al. [15] demonstrated that ResNet outperformed VGG16 in classifying large-scale medical images, including X-ray, MRI, CT, and PET scans from various body regions.

While active research using CNNs has been conducted in the field of image classification, and the classification performance of the models has shown good results, research on image classification using deep learning networks requires considerable time and computing power to optimize numerous parameters in the process of training and classifying large amounts of data. To solve these problems, researchers are actively studying transfer learning. Transfer learning fine-tunes a model trained on a large amount of data in advance according to the problem dataset to be solved, then uses it after optimizing the hyperparameters. In this way, the problem can be effectively solved even when the training data size is small, while reducing the model training time and cost [8]. Seo et al. [16] conducted a fashion item category classification study by using transfer learning. After fine-tuning the last fully connected layer of the GoogLeNet network, pre-trained from many images to their own dataset, category classification was performed. Through transfer learning, it showed good performance even with no labels and a small number of datasets, while simultaneously preventing overfitting with fast learning. Xia et al. [17] applied the self-attention function to the classification layer of a pre-trained Inception-v3 [18] model. By merging three inception modules, each of which extracted different features, the performance of the model increased, and good performance was demonstrated in fashion item classification.

Transformer-based models are also utilized for image classification. Dosovitskiy et al. [19] introduced Vision Transformer (ViT), which processes images by dividing them into 16 x 16 pixels patches and training on these sequences in order. ViT exhibited less inductive bias compared to traditional CNN, which allows users to deal with more general image classification problems when training on enormous datasets. ViT-based classification models have been widely applied in various domains, including classifying brain tumors [20]. However, we did not employ ViT as an image classification model in this project because our research focuses solely on specific types of products, namely fashion items.

### Text-based classification

Text-based category classification is a traditional problem in the field of natural language processing (NLP). Category classification is being developed and applied in various ways. It is used in spam advertisement classification, sentiment analysis, and question-and-answer chatbot technology. Because text data comes from most of our online activities, we can obtain vast amounts of data. However, it is difficult to refine text data, which is unstructured natural language, and extract and utilize features [21]. Traditionally, to utilize and classify text data, a method of directly extracting and predefining the characteristics of the data and classifying the text using a classifier is used. The most famous method of pre-defining text features is the bag of words (BoW) [22] method. Words are manually defined in the word bag, and word categories are then classified using classifiers such as Naive Bayes [23] and support vector machines (SVM) [24]. This method reflects domain knowledge in the process of manually extracting word features and shows good performance in problem solving and text classification of related domains.

However, it does not exhibit good performance in unrelated domains. In addition, because it is a digitization method using the frequency of words, the meaning of words cannot be taken into consideration. As an alternative, latent semantic analysis (LSA) [25], which draws out the latent meaning of words by reducing the dimension, has been proposed. However, LSA is a method of pre-calculating and defining the relationship between words using singular value decomposition (SVD). When a new word is added, the relationships between all words must be recalculated from the beginning. In other words, it can be difficult to reflect new information when it appears [26]. To overcome these disadvantages, Word2Vec [27] has been proposed. This approach uses distributed representation, which is a method of vectorizing the meaning of words in a multidimensional space. In this way, it is possible to express significant similarity between word vectors. In addition, because the meaning between words is identified based on an artificial neural network, new information can be added through additional learning to an existing vector to add information about a new word. However, because Word2Vec, like LSA, defines a corpus of words in advance and utilizes fixed embedding, words that are not in the corpus cannot be output, resulting in an out-of-vocabulary (OOV) problem [28]. Embeddings from Language Model (ELMo) [29], which reflects the context along with the relationships between words, has also been studied. ELMo leverages LSTM [30] to obtain context from a bidirectional pre-trained language model. This solves the problem that Word2Vec and LSA cannot distinguish between homonyms and polyonyms [31]. Subsequently, pre-learning models that trained a large amount of text based on transformers appeared. Bidirectional Encoder Representations from Transformers (BERT) is a pre-trained model released by Google in 2018. BERT is a language model trained on 2.5 billion words from Wikipedia (https://www.wikipedia.org/) and 800 million words from BookCorpus[32], for a total of 3.3 billion words. BERT text data preprocessing reflects the context through contextual embedding, and the OOV problem is solved using the WordPiece tokenizer [33]. In addition, position embedding is used to reflect the sequence of words to solve existing problems. Pre-trained language models learn common language features by learning large amounts of common text data. Subsequently, fine-tuning is performed, in which pre-learned weights are additionally trained according to tasks such as text classification, text summarization, and natural language inference (NLI) [34], and the parameters are readjusted and used. By using pre-training and fine-tuning, the accuracy can be increased without spending large amounts of time and incurring huge costs to learn large amounts of data [35]. Ozyegen et al. [36] conducted an e-commerce product category classification study using Cross-lingual language models (XLM) [37], Vanilla BERT, and Robustly Optimized BERT Pretraining Approach (RoBERTa) [38] models using product title data, and the pre-trained Vanilla BERT model showed good performance compared to the other models. John et al. [39] discussed the recent trends in text-based classification models, finding that BERT-based models remain competent despite its relatively simple structure compared to more recent models like LLaMA[40]. According to the survey, as of 2024, there are two BERT-based models specialized in multilabel text classification among the 151 datasets listed on Papers With Code[41]. One model [42] focuses on analyzing propaganda types in China, while the other [43] is designed for classifying patent types.

### Multimodal-based classification

Multimodal classification refers to the task of classifying data into different types such as image-text and audio-video together. Multimodal classification is being studied in various domains, such as satellite imagery, biometrics, medicine, and product classification. As various datasets and data collection methods have recently been developed, an environment that can be used for multimodal research is being created. Most existing classification algorithms were created by considering a unimodal dataset to process one type of data, such as text, images, and numbers. However, most problems involve a mixture of various data types. Therefore, the overall information cannot be reflected when classification is performed using only one type of data. The problem of classifying data of two different modalities together is difficult, unlike using one type of data in a single modality. In particular, the problem of using the order and time identically (time-synchronized) while using different forms together makes multimodal classification difficult [35]. There are two methods for merging two different modalities into one: early fusion and late fusion. In the early fusion method, the data of each modal is preprocessed, and then each column is created. These results are then used as the input data for the classification model. The late fusion method extracts features from each modality, combines them into one vector, and then classifies them using a classifier. The advantage of the late fusion method is that it selects an algorithm suitable for the characteristics of each modality in the training process, trains each separately, and combines them at the end; thus, it has the advantage of easy feature extraction and training of each modality. Song et al. [44] used late fusion method to classify breast cancer images. Four types of pictures taken from different angles and methods were trained in the ResNet50 model, then features were extracted, output nodes were concatenated, and the final classification was performed. Connecting and using output nodes means that the characteristics of different photos are used together as a single piece of information. To classify breast cancer, the accuracy of the classification can be increased if information taken from different angles and types is reflected and used together, rather than only using information from one angle and type. Sales et al. [45] applied multimodal deep learning to classify e-commerce categories. In the image modality that uses product photos, the VGG16 model was used by applying transfer learning, and in the text modality that uses product description information, the CNN-BiLSTM architecture, which does not use transfer learning, was used. After extracting the features from each modality, the products were classified using the late fusion method. Padi et al. [46] studied emotion analysis by using text and audio data simultaneously and recognizing four emotions. A pretrained BERT was used for the text data modality, and a pretrained ResNet50 was used for the audio data modality. ResNet50 is a model mainly used in the field of computer vision, but it has shown good results in speaker recognition research in recent studies. After the transfer learning of each modality using BERT and ResNet50, text and audio data were combined through the late fusion method, and sentiment analysis was performed through a classifier, which showed better results than those using a single modality when tested by IEMOCAP [47] dataset. Dixit et al. [48] proposed an enhanced emotion recognition model that simultaneously utilizes text, audio and image data to classify six emotions. The model employs a pre-trained text model based on FastText [49] for text-classification, one dimensional (1D) CNN-based multimodal models. Text, audio and image data are integrated using the late fusion with stacking, while mitigating individual model bias, as demonstrated on the CMU-MOSEI dataset [50].

Several researchers have further demonstrated that multimodal classification models outperform unimodal classification models [51,52]. Zou et al. [51] introduced the Unimodality-Supervised MultiModal Contrastive Learning (UniS-MMC) model, a multimodal classification model based on contrastive learning, evaluated on two image-text classification benchmarks, UPMC-Food-101 [53] and N24News [54]. Villegas et al. [52] presented a multimodal classification model for social media posts, which is an integration of Bernice [55] based text model and ViT based image models. Their model incorporates image-text contrastive learning or image-text matching techniques to capture the relationship between images and texts in the posts. Five diverse image-text multimodal datasets [56–60] are benchmarked. Table 1 shows the overview of multimodal classification models described in the paper so far.

**Table 1:** The literature review of multimodal classification.

Study	Base Classification Model	Target Objects	Fusion Method	Key Features/Objective
Song et al. [44]	MVMM-Net, Combination of CNN-based image classification models like ResNet.	Four X-ray images of two different angles and processes (LE/DES).	Late Fusion	Classifying breast cancer images to determine whether tumors are benign or malignan.t
Sales et al. [45]	VGG image classification model and CNN + BiLSTM-based text classification model.	E-commerce images and description of products.	Late Fusion	Using classification model to manage e-commerce catalog.
Padi et al. [46]	Pre-trained ResNet-based audio recognition model and Fine-tuned BERT based text classification model using transfer learning	Audio and text dataset describing a kind of emotion.	Late Fusion	Performing emotional recognition from simultaneous audio and text dataset. Using IEMOCAP [47] dataset for benchmarking.
Dixit et al. [48]	1D CNN-based audio recognition model, 2D CNN-based image recognition model and FastText[49] text classification model.	Image, audio and text dataset describing a kind of emotion.	Late Fusion	Performing emotional recognition from simultaneous text, audio and image dataset. Using CMU-MOSEI []50] dataset for benchmarking.
Zou et al. [51]	UniS-MMC. Combination of ViT based image classification model and BERT-based text model, with using supervised multimodal contrastive learning.	Image and Text paired dataset, with text matching mutually matching images.	Early Fusion	Classifying various multimodal data. Using UPMC-Food-101 [53] and N24News [54] dataset for benchmarking.
Villegas et al. [52]	Combination of Bernice[55] based text-classification model and ViT based image classification model.	Social Media Posts with pairs of images and texts.	Late Fusion	Classifying social media posts. Using five diverse multimodal public datasets in English: TIR [56], MVSA [57], MHP [58], MSD [59], MICD [60]

**Table 2:** Fashion Product Images Dataset example.

id	gender	category	base color	season	year	usage	product Display Name	file name
15970	M	Shirts	Navy Blue	F	2011	Casual	Turtle Check Men Navy Blue Shirt	15970.jpg
39386	M	Jeans	Blue	s	2012	Casual	Peter England Men Party Black Jeans	39386.jpg
59263	W	Watches	Silver	W	2016	Casual	Titan Woman Silver Watch	59263.jpg
21379	M	Track Pants	Black	F	2011	Casual	Manchester United Men Solid Black Track Pants	21379.jpg

## Method

### Data collection and exploration

**Fig 1:**
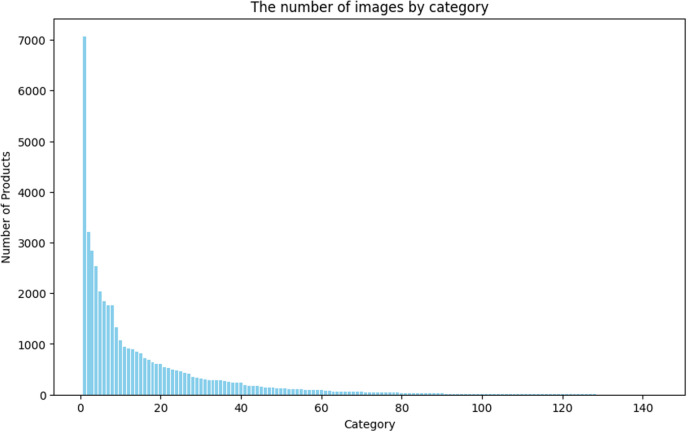
The number of images by category for Fashion Product Images Dataset.

In this study, we used the Fashion Product Images Dataset [61] provided by Kaggle (https://www.kaggle.com/). It consists of 15.71 GB, 11 columns, and 44,416 data. Table 2 presents an example of a dataset column. The image data are high-resolution images of different sizes, 1080 × 1440 or 1800 × 2400, in the form of a JPG file. Among various types of data, the data used in this study used actual product image files and product display names to classify product categories such as shirts, jeans, watches, and trousers. Fig 1 shows the number of products by category. Owing to the nature of fashion e-commerce data, the skewness of the number of products per category is severe at 5.611. In addition, among the 143 categories, the category with the largest number of products is the T-shirt category, which has 7,065 data, and the category with the smallest number has one datum. If the data exhibits an imbalanced distribution, the imbalance can hinder model learning.

### Features of fashion e-commerce data

Compared to general e-commerce product photos, fashion e-commerce product photos depict models wearing products, sometimes causing misclassification. There is an image of a product of the cap category in the dataset with a male model. however, the model’s face is more prominent than the cap. There is also an image of a product with a female model wearing a dress, categorized as dress category, but the model is wearing the dress along with a bag and sunglasses, which are products of other categories. Contrary to images of fashion e-commerce products, e-commerce images of electronic devices contain only the images of the products and do not include images of a model or other products. Compared to other e-commerce categories, the feature of fashion e-commerce product image data is that a model is shown wearing multiple products together, making it difficult to recognize the product in that category. Therefore, difficulties arise when classifying product categories using only image data.

### Data processing

The dataset has an unbalanced distribution by category. An under-sampling method was applied to solve the asymmetry problem by category. Among a total of 143 categories, the 39th largest category, which name is Kurtis and contains 234 items, is essentially the same category as the 6th largest category named Kurtas. Therefore, we set the threshold of at least 250 items to exclude products that belong to essentially the same category and reduce misclassification. There are 106 categories in which the number of product data is less than 250 and thus excluded from the dataset. Also, largest categories like t-shirt contain over a thousand or even several thousand items, but since many of these items are highly similar, differing only in minor aspects such as color or pattern, selecting 250 items at random is unlikely to result in a significant loss of diversity information. Therefore, we performed under-sampling by randomly selecting 250 items from each of the remaining 37 categories to address the performance degradation issue caused by the imbalance in category sizes. Of the 250 items in each category, 175, 25, and 50 were used for training, verification, and testing, and the dataset was constructed with a ratio of 7:1:2. Including all 37 categories’ data, the final dataset consisted of 6,475 images and text entries for training, 925 for verification, and 1,850 for testing.

Image and text data were preprocessed according to ResNet50 and BERT, which were used as the backbone networks for each modality. In the image data pre-processing of fashion items, the size of the original image data was 1080 × 1440 or 1800 × 2400, depending on the item. To solve this problem, the first step was to change the size of all images to be equal, using a size of 256 × 256, which is the input size of the ResNet50 model. Next, the final pre-processing was performed with a size of 224 × 224 centered on the center of the image, and the size was converted to be the same as the input size of the ResNet50 architecture. Special characters, stop words, and spaces were removed from the product title text data. Then, each sentence was tokenized using the BERT Tokenizer provided by PyTorch [62], and [CLS] and [SEP] tokens were added to indicate the start and end of the sentence. Next, it was padded to a length of 30 characters, which was the maximum length of the product title data. Padding is a process in which words of less than 30 characters are filled with as many zeros as the number of remaining characters to match the target length. In the next step, position encoding was performed to generate sequence information by converting a sequence of sentences into a token ID. Finally, we created an attention mask. The product images and title data that underwent preprocessing were used as input data for each modality.

### Research model and optimization experiment strategies

#### Research model.

**Fig 2:**
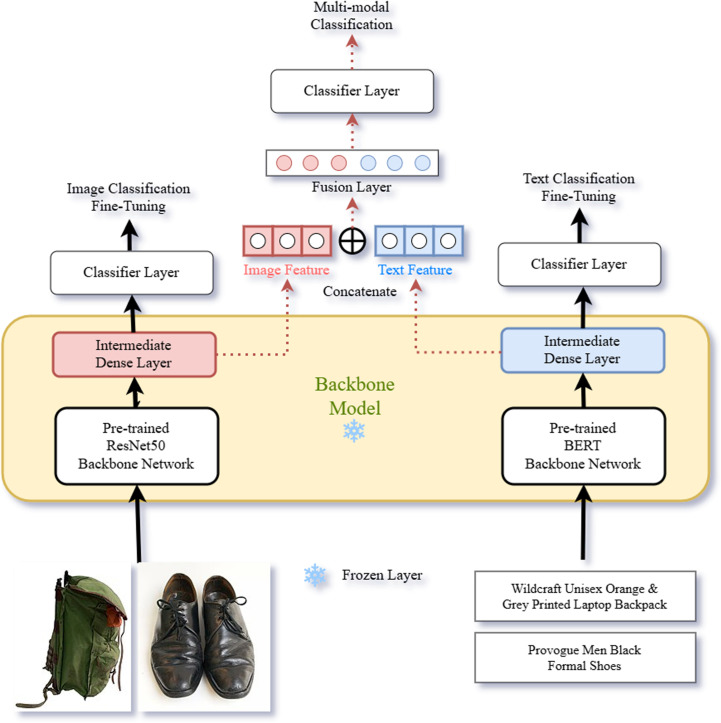
Fashion e-commerce multimodal classification model structure.

Fig 2 shows the architecture of the proposed optimization model that classifies fashion products using both image and text data. The model mainly consists of three steps. The first step uses pre-trained models to fine-tune each image module and text module to match the fashion e-commerce product images and text data. The image module employs a pre-trained ResNet50, provided by PyTorch [62], as its backbone network, while the text module utilizes the pre-trained BERT-base-uncased model from Hugging Face [63] as its backbone network. The second step freezes the fine-tuned image and text modules so that the model no longer learns. After feature vectors of images and text are extracted from each module, they are concatenated and fused into one vector. In the last step, the fusion dense layer and classifier layer are fine-tuned, and product categories are classified.

### Model optimization experimental strategy

To design a model optimized for the fashion e-commerce dataset, we explored an optimized strategy by applying transfer learning techniques and multimodal model convergence methods in various ways. In the process of fine-tuning optimized fashion e-commerce data by importing pre-trained models of image and text modules, experiments were conducted by dividing the degree of freezing of part of the neural network into three strategies to adjust the weights of all or only part of the neural network. The experiment was conducted by dividing the multi-modal fine-tuning method, which combines the image and text modules, into six strategies. In addition, we compared five fusion methods that fuse image and text modules.

### ResNet50 Backbone Network transfer learning

**Fig 3:**
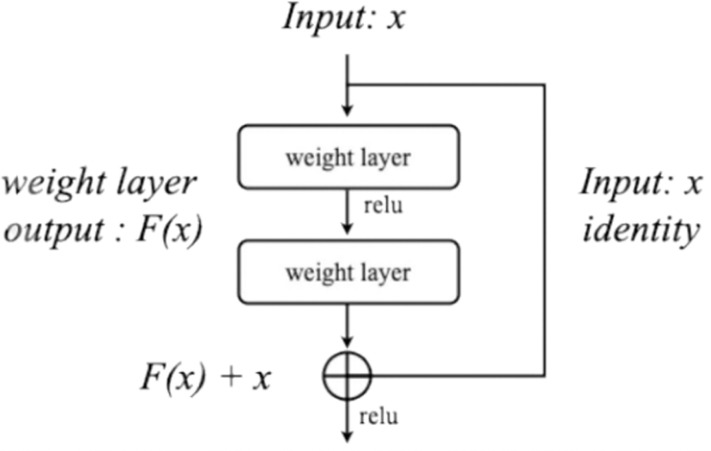
Structure of residual learning.

ResNet is widely used as a backbone network across various computer vision applications, serving as a framework model for solving problems such as classification, object detection, image segmentation, and pose estimation. The process of extracting image features is essential for these tasks, and ResNet is primarily employed for this purpose. To address the vanishing gradient problem that occurs when the network depth increases, ResNet uses residual learning. Residual learning reuses the results of previous layers [11]; its structure is shown in Fig 3. The input x passes through a weight layer and an activation function, and the value F(x) is the output. By adding the input x again, a final output value of F(x)+x is calculated and used as the input value of the next layer. The ResNet50 model utilized a pre-trained model with ImageNet, a large-scale dataset provided by PyTorch [62]. The images included in the entire dataset consist of more than 10 million data and 1,000 classes [64], and the final dense layer of the ResNet50 architecture outputs in 1,000 dimensions. After adjusting the output dimension of the last dense layer to 500 according to the classifier and dataset used in this study, it was used as the input value of the classifier. The classifier received a 500-dimensional vector, output a 37-dimensional probability (which is the number of classes in the dataset used in this study), and classified the data. ResNet50, a backbone network that extracts features from image data, performed an optimization process using the product photos of the Fashion Product Images Dataset used in this study as training data. Cross entropy was used as the fine-tuning loss function, and Adam [65] was used as the optimizer. In addition, random horizontal flip, a data augmentation technique, was applied to the training data. The ResNet50 optimization fine-tuning process consists of the following four steps:

• **First step:** Transfer and utilize the architecture and weights of the pre-trained ResNet50 model.
• **Second step:** Freeze the pre-trained ResNet50 model to prevent further training.
• **Third step:** A classifier composed of a Dense layer-ReLU-Dropout-Dense layer is added to the ResNet50 architecture.
• **Last step:** The classifier is trained and fine-tuned using fashion product image data.

### BERT backbone network transfer learning

**Fig 4:**
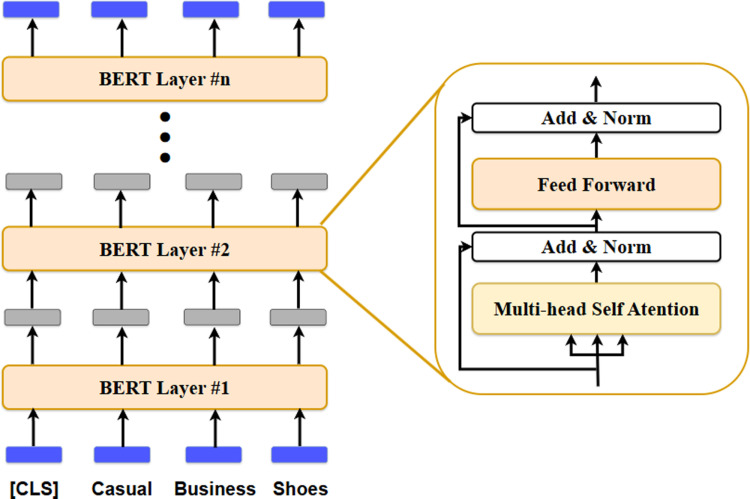
BERT architecture consisting of transformer’s encoder.

BERT trained with large amounts of data exhibits good performance in various NLP problems and is used as a backbone network through transfer learning. Four main types of problems can be solved using BERT: sentence classification, sentence pair classification, single sentence tagging tasks, and question and answering. Various problems can be solved through a fine-tuning process, in which additional layers are added to the final output layer of the BERT model according to the task to be solved, and the parameters are readjusted along with additional training with a new dataset. Fig 4. shows the basic structure of BERT, in which the transformer encoders are stacked several times. The BERT layers perform self-attention and position-wise feedforward neural networks.

The pre-trained BERT model utilized the BERT-base-uncased model provided by Hugging Face [63]. The BERT-based model can be used with English data and includes 12 layers of BERT composed of transformer encoders. The BERT-base-uncased model can be used in both uppercase and lowercase English languages. BERT’s last dense layer outputs 768 dimensions. An intermediate dense layer that receives the last output layer as an input value is additionally configured in the classifier, received as a 768-dimensional input, reduced to 500 dimensions, and used as an input value for the final classifier layer. After receiving a 500-dimensional vector, the classifier finally outputs a 37-dimensional probability and classifies the data. BERT, a backbone network that extracts features from text data, underwent an optimization process using the English product titles of the Fashion Product Images Dataset as training data. The fine-tuning training loss function uses cross entropy, which is used in classification problems. AdamW [66] was used as the optimizer. To prevent the problem of exploding gradients, which frequently occurs in the training process of BERT, gradient clipping was applied, and the gradient was set so that the gradient did not exceed 1. Without clipping, the gradient vector may not reach its global minimum during gradient descent and may progress in the wrong direction. When clipping was applied, the gradient vector was stably learned by moving a small value while maintaining its direction. BERT’s optimization fine-tuning process of the BERT consists of the following three steps.

• **First step:** Transfer and utilize the architecture and weights of the pre-trained BERT model.
• **Second step:** A classifier consisting of a Dense layer-ReLU-Dropout-Dense layer is added to the BERT architecture.
• **Last step:** Use the fashion product title data to train and fine-tune the entire model.

### Image and text module transfer learning strategies

**Fig 5:**
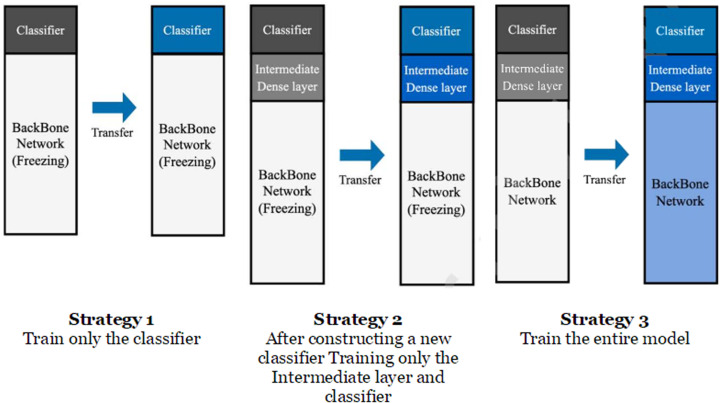
Transfer learning strategies for image and text modules.

To determine the best optimization transfer learning method for each image and text module, we compared three strategies for optimizing each module for the fashion e-commerce dataset. In the process of fine-tuning the backbone network in transfer learning, the transfer learning strategy of freezing a part of the neural network to adjust and optimize the level of updating all or some weights of the neural network is divided into three strategies, as illustrated in Fig 5.

- **Strategy 1:** Train and use only the classifier after freezing so that the feature extraction backbone layer is not updated. The weights of the pretrained model are not updated, but used as they are, and only the classifier part is trained according to this research dataset.
- **Strategy 2:** Delete the existing classifier and construct a new intermediate dense layer and classifier. After feature extraction, the backbone layer is frozen so that it is not updated, and only the newly built intermediate dense layer and classifier are trained according to this research dataset.
- **Strategy 3:** This is a strategy for learning the entire model. The model is trained starting from the weights of the pretrained model. The weights of the entire model are updated from the backbone layer to the newly configured classifier through additional training.

### Multi-modal classifier fine-tuning

The final category classification was carried out using a multimodal classifier that combines features extracted from image and text data. The fine-tuning sequence of the final classifier consisted of five steps. Fig 2. shows the architecture of an optimized multimodal classification model that performs classification by fusing the features of image and text data.

• **First step:** The architecture and weight of the image and text modules that were fine-tuned according to the dataset used in this study are transferred and used.
• **Second step:** Freeze the model from the fine-tuned image and text modules to prevent further training.
• **Third step:** Image and text feature vectors are extracted from the intermediate dense layer, which is a hidden layer before the classifier of each module.
• **Fourth step:** Fuse the two feature vector outputs from the middle dense layer of each module.
• **Last step:** Fine-tune the final classifier consisting of a Fusion layer-ReLU-Dropout-Dense layer by training it with fashion product images and fashion product title training data.

The middle dense layer, which extracts the features of each image and text module, outputs 500 dimensions. In the fusion layer, the two feature vectors are fused to receive a 1000-dimensional input and reduce the dimensions to 250. Subsequently, the final classifier layer receives a 250-dimensional input value, outputs a 37-dimensional probability, and classifies the data. Cross entropy was used as the fine-tuning training loss function of the final classifier. AdamW was used as the optimizer. In addition, to prevent the problem of exploding gradients, gradient clipping was applied, and the gradient was set such that it did not exceed 1.

### Multimodal model fine-tuning strategy

**Fig 6:**
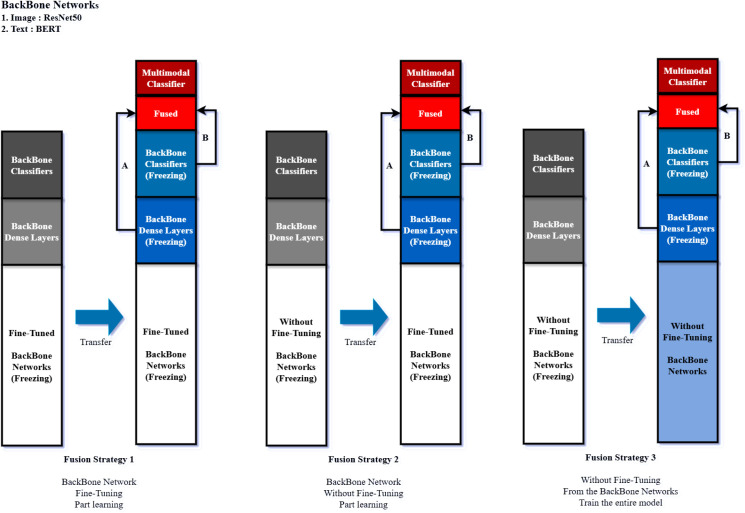
Fine-tuning Strategy for Multimodal Models.

Now we compare the effectiveness of fine-tuning the backbone models. Fig 6. shows three fusion strategies, with or without fine-tuning the backbone models, or usage of partial training or entire training. Each strategy integrates with middle dense layers or middle classifier layer.

• **Fusion Strategy 1-A:** Classify the image and text features after extracting the image and text features from the middle dense layer of each module after freezing the optimized fine-tuned image and text module backbone networks according to the dataset of this experiment so that they are not updated.
• **Fusion Strategy 1-B:** Extract image and text features from the classifier layer of each module after freezing the optimized fine-tuned image and text module backbone networks according to the dataset of this experiment and classify them through fusion.
• **Fusion Strategy 2-A:** After freezing the transfer-learned image and text module backbone networks without finetuning so that they are not updated, extract image and text features from the middle dense layer of each module and fuse them to classify them.
• **Fusion Strategy 2-B:** After freezing the image and text modules, backbone networks are transferred without finetuning so that they are not updated; the image and text features of the classifier layer of each module are extracted, then fused and classified.
• **Fusion Strategy 3-A:** Start with the backbone network of transfer-learned image and text modules without finetuning, learn the entire model, extract image and text features from the middle dense layer of each module after finetuning, and then fuse and classify them.
• **Fusion Strategy 3-B:** Learn the entire backbone network of image and text modules transferred from scratch without fine-tuning, extract image and text features from the classifier layer of each module after fine-tuning, and fuse and classify them.

### Fusion methodology

To compare the model optimization performance according to the fusion method of the image and text feature vectors, five fusion methods were compared. The fusion of image and text data is expressed as $D={\left(r_{i},s_{i}\right)}_{i=1:n}$, where $r_{i}$ denotes the ith image, and $s_{i}$ the ith text. The feature vectors extracted from each modality were equally matched to 500 dimensions. The feature vector extracted from the image modality can be expressed as $r_{i}\in R^{\left(500\right)}$, and the feature vector extracted from the text modality can be expressed as $s_{i}\in R^{\left(500\right)}$. The first fusion method is the addition of feature vectors of two modalities, and is expressed as follows:


$$D=r_{i}\in R^{\left(500\right)}+s_{i}\in R^{\left(500\right)}$$ (1)

The second fusion method calculates the average of the feature vectors of two modalities, and is expressed as follows:


$$D=\left(r_{i}\in R^{\left(500\right)}+s_{i}\in R^{\left(500\right)}\right)/2$$ (2)

The third fusion method calculates the element-wise product of the feature vectors of two modalities, and is expressed as follows:


$$D=r_{i}\in R^{\left(500\right)}\;⨀s_{i}\in R^{\left(500\right)}$$ (3)

The fourth fusion method concatenates feature vectors of two modalities, and is expressed as follows:


$$\begin{array}{c} D=\text{concat}\left(r_{i}\in R^{\left(500\right)},\;s_{i}\in R^{\left(500\right)}\right) \end{array}$$ (4)

The last fusion method fuses image and text vectors by passing only important information using a gating mechanism. σ denotes the sigmoid function, which is the activation function. $g_{t}$ represents the gate network, and outputs the result of passing through the gate as 0 or 1. A value of 1 passes information completely, and 0 completely discards information so that it is not passed to the input value of the next layer. ⊙ represents an element-wise product. If $g_{t}$ is set to 0, features extracted from the image modality are ignored, and the feature vectors extracted from the text modality are passed to the next layer. When $g_{t}$ is set to 1, features extracted from the text modality are ignored, and feature vectors from image modalities are passed to the next layer.


$$\begin{array}{c} x_{i}=\text{concat}\left(r_{i}\in R^{\left(500\right)},\;s_{i}\in R^{\left(500\right)}\right) \end{array}$$ (5)

$$g_{t}\;=\;\sigma (x_{i}+b)$$ (6)

$$D=(g_{t}⨀r_{i}\in R^{\left(500\right)})$$ (7)

## Experiments and results

### Experimental environment

The experimental environment for the model in this study included Python 3.8, PyTorch 1.7.1, and an NVIDIA GeForce RTX 3060 GPU.

### Experiment results

After applying the step-by-step optimization method, the optimization method that showed the best performance was identified. In addition, the performance of the final model was compared with that of image and text models using only single data. To evaluate the optimization performance, we present the training time at which the model converged along with its accuracy. In addition, the numbers of trainable and non-trainable parameters according to each optimization method are presented.

**Fig 7:**
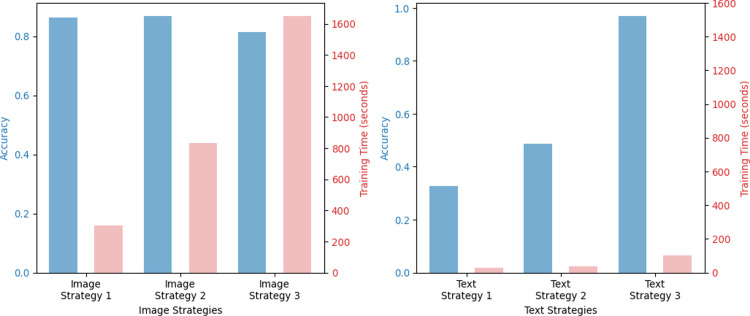
Image model and text model optimization strategy experiment results.

### Image model optimization strategy experiment results

An image model optimization experiment was conducted by applying the fine-tuning strategy suggested in Section 3.4.5. As shown in Table 3, Strategy 2, which creates a new classifier layer and an intermediate dense layer and trains the backbone network after freezing, showed the highest accuracy. As the middle dense layer was added, the number of fully connected layers increased; therefore, the number of trainable parameters increased compared to Strategy 1, which increased the training time of the model. The left graph in Fig 7 presents our results about image model optimization strategy, with the left bars indicating accuracy and the right bars denoting training time.

**Table 3:** Image model optimization strategy experiment results comparison.

	Accuracy	Training time	Total parameters	Trainable parameters	Non-trainable parameters
Strategy 1	0.8632	5m 1 s	23,583,845	75,813	23,508,032
**Strategy 2**	**0.8697**	**13m 55s**	**26,076,069**	**2,568,037**	**23,508,032**
Strategy 3	0.8146	27 m 32s	26,070,069	26,076,069	0

### Experimental results of text model optimization strategies

Table 4 lists the results of applying the fine-tuning strategy presented in Section 3.4.5. to the text model. Strategy 3 showed the highest accuracy. Strategy 3 creates a new classifier layer and an intermediate dense layer and trains the entire model. The transfer learning of the BERT model showed good performance, even though it had a relatively short training time. The right graph in Fig 7 also presents our results about text model optimization strategy, with the left bars indicating accuracy and the right bars denoting training time.

**Table 4:** Comparison of text model optimization strategy experiment results.

Strategy	Accuracy	Training time	Total parameters	Trainable parameters	Non-trainable parameters
Strategy 1	0.3276	27s	109,510,693	28,453	109,482,240
Strategy 2	0.4859	36s	109,885,277	403,037	109,482,240
**Strategy 3**	**0.9708**	**1m 42s**	**109,885,277**	**109,885,277**	0

**Fig 8:**
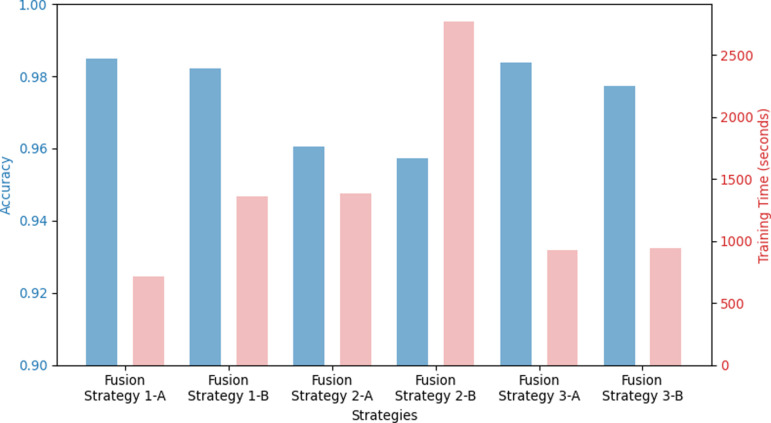
Multimodal model optimization strategy experimental results.

### Multimodal model optimization strategy experimental results

A multimodal model optimization experiment was conducted by applying the fine-tuning strategy suggested in Section 3.4.7. The multimodal fusion method was tested based on the method of concatenating the feature vectors of the image and text modules. As shown in Table 5, Fusion Strategy 1-A converged with the best performance and a relatively short training time. The 1-A strategy utilizes pre-optimized models for each image and text module as a backbone network. The pre-optimized backbone network is a strategy for extracting and fusing features from the middle dense layer of each module after freezing the entire network so that it is not updated. Compared to the 1-B strategy, which extracts and fuses features from the final classifier layer, it converged approximately twice as fast and showed better performance. Fig 8 also presents our result, with left bar indicating accuracy and right bar indicating training time.

**Table 5:** Multimodal Model Optimization Strategies Comparison of Experimental Results.

Strategy	Accuracy	Training time	Total parameters	Trainable parameters	Non-trainable parameters
**Fusion** **Strategy 1-A**	**0.9849**	**11 m 50 s**	**136,220,883**	**259,537**	**135,961,346**
Fusion Strategy 1-B	0.9822	22 m 43 s	135,969,671	8,325	135,961,346
Fusion Strategy 2-A	0.9605	23 m 7 s	36,220,883	1,181,611	135,039,272
Fusion Strategy 2-B	0.9573	46 m 14s	135,969,671	930,399	135,039,272
Fusion Strategy 3-A	0.9838	15 m 28 s	136,220,883	136,220,883	0
Fusion Strategy 3-B	0.9773	15 m 40 s	135,969,671	135,969,671	0

### Fusion methodology experiment results

The results of the five fusion methods presented in Section 3.5 were compared. The fusion methods were tested by changing them based on Fusion Strategy 1-A. As a result of the experiment in Table 6, the concatenation method showed the best performance. Concatenation is a method that does not lose information by connecting two feature vectors. However, the gate network method leaves only meaningful information among text and image features and loses the rest of the information, resulting in information loss. In addition, element-wise, average, and addition methods reduce information by calculating text and image information and combining them into one piece of information. Therefore, the concatenation method, which does not lose information, showed the best performance.

**Table 6:** Comparison of fusion methodology experiment results.

Fusion method	Accuracy	Training time	Total parameters	Trainable parameters	Non-trainable parameters
Addition	0.9843	11 m 49 s	136,095,883	134,537	135,961,346
Average	0.9832	11 m 49 s	136,095,883	134,537	135,961,346
Element-wise	0.9838	11 m 52s	136,095,883	134,537	135,961,346
Gate network	0.9838	11 m 54s	136,596,383	635,037	135,961,346
**Concatenate**	0.9849	**11m 50s**	**136,220,883**	**259,537**	**135,961,346**

**Fig 9:**
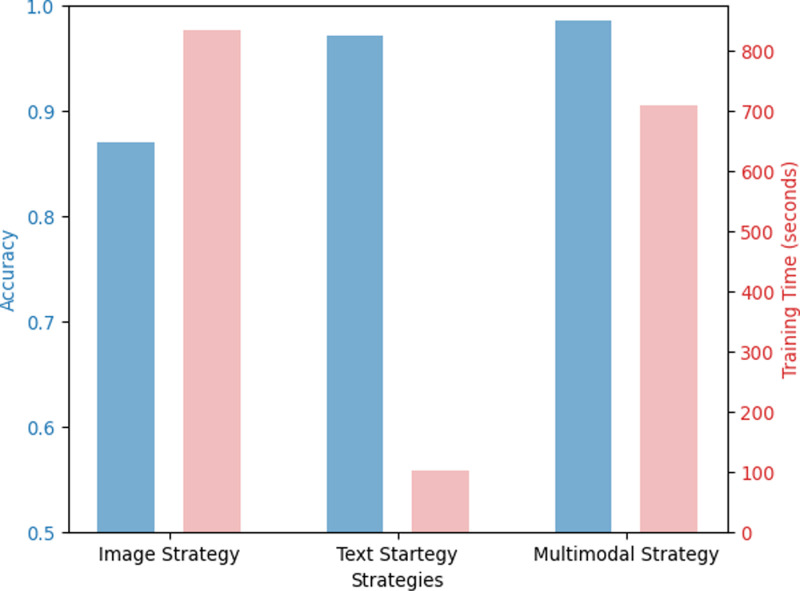
Comparison of optimized unimodal and multimodal experimental results.

### Comparison of unimodal and multimodal models

Next, we compared the effectiveness of multimodal and unimodal models. The image model was fine-tuned using Strategy 2, and the text model was fine-tuned using Strategy 3; these were compared with the optimized fine-tuned model for each model. The multi-modal model is optimized and fine-tuned with Fusion Strategy 1-A proposed in this study. The multimodal models reduce about 50% error rate compared to text model and one seventh of error rate compared to image model as shown in Table 7. Fig 9 also shows the visualization of our results, with left bar indicating accuracy and right bar indicating training time.

**Table 7:** Comparison of optimized unimodal and multimodal experimental results.

Modal	Accuracy	Training time	Total params	Trainable params	Non-trainable params
Image modal	0.8697	13 m 55s	26,076,069	2,568,037	23,508,032
Text modal	0.9708	1 m 42 s	109,885,277	109,885,277	0
**Multi-modal**	**0.9849**	**11 m 50 s**	**136,220,883**	**259,537**	**135,961,346**

## Conclusion and future work

In this study, a method for optimizing fashion e-commerce product classification based on multimodal and transfer learning was presented. Through the proposed methodology, the bias of data, which is a problem in e-commerce fashion product category classification, was solved using the under-sampling method. Next, to overcome the problem of low-quality fashion e-commerce data with noise, we applied a multimodal deep learning method that simultaneously learns different types of data to provide more information to the model to reduce the influence of noise. In addition, the problem of requiring a large amount of computing resources and a long learning time when simultaneously training a large number of image and text data was solved through transfer learning. Finally, the problem of model performance degradation resulting from the reduced amount of data during the under-sampling process was solved through transfer learning of the pretrained model. As for practical implications, a transfer learning strategy and optimization fine-tuning method for each image and text module were presented, and a total of 14 methods for the fusion of image and text modules and optimization methods for multimodal models were presented. Multimodal deep learning can reflect more information in a deep learning model compared to using only a single dataset with a single modality. Thus, based on a relatively large amount of information, the model showed good performance compared to single-modal models. Referring to the transfer learning optimization fine-tuning strategy and multimodal model construction methodology presented in this study, it is expected that practitioners will be able to build an optimal model by selecting the appropriate method for the actual business environment, in which fast learning and inference are important.

To further develop the results of this study, we need to consider how to train models for other categories with fewer items. This study focused on 37 categories with abundant items among the 143 categories in the e-commerce market. Thus, to classify the remaining categories, methods such as oversampling of smaller categories may be necessary. Additionally, the study tested the model using selected items through under-sampling rather than using the original raw data. Therefore, it is important to evaluate the possibility that the models developed in this study may be overfitting to the training data. Despite these limitations, this study provides an effective method for categorizing e-commerce products by combining image-based and text-based classification models.

## References

[1] Statistics Korea. Press release on online shopping trends in Korea [Internet]. Available from: https://kostat.go.kr/board.es?mid=a10301120300&bid=241&act=view&list_no=433999

[2] NAVER Corp. DEVIEW blog post [Internet]. Available from: https://d2.naver.com/helloworld/1264836#eng s

[3] Yeon H. Musinsa, Ably, and Zigzag soar as top 3 fashion apps hit record sales [Internet]. Money S Article. Available from: https://www.moneys.co.kr/article/2024040915522182917

[4] Yen S-J, Lee Y-S. Cluster-based under-sampling approaches for imbalanced data distributions. Expert Systems with Applications. 2009;36(3):5718–27. doi: 10.1016/j.eswa.2008.06.108

[5] Anand A, Pugalenthi G, Fogel GB, Suganthan PN. An approach for classification of highly imbalanced data using weighting and undersampling. Amino Acids. 2010;39(5):1385–91. doi: 10.1007/s00726-010-0595-2 20411285

[6] Inoue N, Furuta R, Yamasaki T, Aizawa K. Multi-label fashion image classification with minimal human supervision. In: Proceedings of the IEEE International Conference on Computer Vision Workshops (ICCVW); 2017. doi: 10.1109/iccvw.2017.265

[7] Kolisnik B, Hogan I, Zulkernine F. Condition-CNN: A hierarchical multi-label fashion image classification model. Expert Systems with Applications. 2021;182:115195. doi: 10.1016/j.eswa.2021.115195

[8] Pan SJ, Yang Q. A Survey on Transfer Learning. IEEE Trans Knowl Data Eng. 2010;22(10):1345–59. doi: 10.1109/tkde.2009.191

[9] Wang W, Sun Y, Wang B, Liu H, Xu S. Development of convolutional neural network and its application in image classification: a survey. Opt Eng. 2019;58(4):040901.

[10] Zhen L, Hu P, Peng X, Goh RSM, Zhou JT. Deep Multimodal Transfer Learning for Cross-Modal Retrieval. IEEE Trans Neural Netw Learn Syst. 2022;33(2):798–810. doi: 10.1109/TNNLS.2020.3029181 33090960

[11] He K, Zhang X, Ren S, Sun J. Deep residual learning for image recognition. In: Proceedings of the IEEE Conference on Computer Vision and Pattern Recognition (CVPR); 2016. doi: 10.1109/cvpr.2016.90

[12] Krizhevsky A, Sutskever I, Hinton GE. ImageNet classification with deep convolutional neural networks. Commun ACM. 2017;60(6):84–90. doi: 10.1145/3065386

[13] Simonyan K, Zisserman A. Very deep convolutional networks for large-scale image recognition. In: Proceedings of the International Conference on Learning Representations (ICLR); 2015.

[14] Szegedy C, Liu W, Jia Y, Sermanet P, Reed S, Anguelov D, et al. Going deeper with convolutions. In: Proceedings of the IEEE Conference on Computer Vision and Pattern Recognition (CVPR); 2015. p.1-9.

[15] Macfadyen C, Duraiswamy A, Harris-Birtill D. Classification of hyper-scale multimodal imaging datasets. PLOS Digit Health. 2023;2(12):e0000191. doi: 10.1371/journal.pdig.0000191 38091333 PMC10718410

[16] Seo Y, Shin KS. Image classification of fine-grained fashion image based on style using pre-trained convolutional neural network. In: Proceedings of the IEEE International Conference on Big Data Analysis (ICBDA); 2018.

[17] Xia T, Zhang J. Clothing classification using transfer learning with squeeze and excitation block. Multimed Tools Appl. 2022;81:22051–68.

[18] Szegedy C, Vanhoucke V, Ioffe S, Shlens J, Wojna Z. Rethinking the Inception Architecture for Computer Vision. In: Proceedings of the IEEE Conference on Computer Vision and Pattern Recognition (CVPR); 2016.

[19] Dosovitskiy A, Beyer L, Kolesnikov A, Weissenborn D, Zhai X, Unterthiner T, et al. An image is worth 16x16 words: transformers for image recognition at scale. arXiv preprint arXiv:2010.11929; 2020.

[20] Hong S, Wu J, Zhu L, Chen W. Brain tumor classification in VIT-B/16 based on relative position encoding and residual MLP. PLoS One. 2024;19(7):e0298102. doi: 10.1371/journal.pone.0298102 38954731 PMC11218980

[21] Minaee S, Kalchbrenner N, Cambria E, Nikzad N, Chenaghlu M, Gao J. Deep Learning--based Text Classification. ACM Comput Surv. 2021;54(3):1–40. doi: 10.1145/3439726

[22] Zhang Y, Jin R, Zhou Z. Understanding bag-of-words model: a statistical framework. Int J Mach Learn Cybern. 2010;1:43–52.

[23] Rish I. An empirical study of the naive Bayes classifier. In: Proceedings of JCAI 2001 workshop on Empirical Methods in Artificial Intelligence; 2001.

[24] Hearst MA, Dumais ST, Osuna E, Platt J, Scholkopf B. Support vector machines. IEEE Intell Syst Their Appl. 1998;13(4):18–28. doi: 10.1109/5254.708428

[25] Deerwester S, Dumais ST, Furnas GW, Landauer TK, Harshman R. Indexing by latent semantic analysis. J Am Soc Inf Sci. 1990;41(6):391–407.

[26] Devlin J, Chang MW, Lee K, Toutanova K. BERT: Pre-training of deep bidirectional transformers for language understanding. In: Proceedings of the North American Chapter of the Association for Computational Linguistics: Human Language Technologies (NAACL-HLT); 2019. p.4171–4186.

[27] Mikolov T, Chen K, Corrado G, Dean J. Efficient estimation of word representations in vector space. arXiv preprint arXiv:1301.3781; 2013.

[28] Horn F. Context encoders as a simple but powerful extension of word2vec. In: Proceedings of the Workshop on Representation Learning for NLP; 2017. p.10–14.

[29] Peters ME, Neumann M, Iyyer M, Gardner M, Clark C, Lee K, et al. Deep contextualized word representations. In: International Conference on Learning Representations (ICLR); 2018.

[30] Hochreiter S, Schmidhuber J. Long short-term memory. Neural Comput. 1997;9(8):1735–80. doi: 10.1162/neco.1997.9.8.1735 9377276

[31] Sarzynska-Wawer J, Wawer A, Pawlak A, Szymanowska J, Stefaniak I, Jarkiewicz M, et al. Detecting formal thought disorder by deep contextualized word representations. Psychiatry Res. 2021;304:114135. doi: 10.1016/j.psychres.2021.114135 34343877

[32] Zhu Y, Kiros R, Zemel R, Salakhutdinov R, Urtasun R, Torralba A, et al. Aligning Books and Movies: Towards Story-Like Visual Explanations by Watching Movies and Reading Books. 2015 IEEE International Conference on Computer Vision (ICCV). 2015. p. 19–27. doi: 10.1109/ICCV.2015.11

[33] Song X, Salcianu A, Song Y, Dopson D, Zhou D. Fast wordpiece tokenization. In: Proceedings of the Empirical Methods in Natural Language Processing (EMNLP); 2021. p.2089–2103.

[34] Bowman SR, Angeli G, Potts C, Manning CD. A large annotated corpus for learning natural language inference. In: Proceedings of the Empirical Methods in Natural Language Processing (EMNLP); 2015. p.632–642.

[35] Sleeman WC IV, Kapoor R, Ghosh P. Multimodal Classification: Current Landscape, Taxonomy and Future Directions. ACM Comput Surv. 2022;55(7):1–31. doi: 10.1145/3543848

[36] Ozyegen O, Jahanshahi H, Cevik M, Bulut B, Yigit D, Gonen FF, et al. Classifying multi-level product categories using dynamic masking and transformer models. J of Data, Inf and Manag. 2022;4(1):71–85. doi: 10.1007/s42488-022-00066-6

[37] Lample G, Conneau A. Cross-lingual language model pretraining. In: Proceedings of the International Conference on Neural Information Processing Systems (NeurIPS); 2019. p.7059-7069.

[38] Liu Y, Ott M, Goyal N, Du J, Joshi M, Chen D, et al. RoBERTa: a robustly optimized BERT pretraining approach. arXiv preprint arXiv:1907.11692; 2019.

[39] John F, Chovanec K, Madiraju P. A survey of text classification with transformers: how wide? how large? how long? how accurate? how expensive? how safe? IEEE Access. 2023;12:6518-31.

[40] Touvron H, et al. LLaMA: open and efficient foundation language models. arXiv preprint arXiv:2302.13971; 2023.

[41] Papers With Code [Internet]. Available from: https://paperswithcode.com/

[42] Lee JS, Hsiang J. PatentBERT: patent classification with fine-tuning a pre-trained BERT model. arXiv preprint arXiv:1906.02124; 2019.

[43] Chang RC, Lai CM, Chang KL, Lin CH. Dataset of propaganda techniques of the state-sponsored information operation of the people’s republic of China. arXiv preprint arXiv:2106.07544; 2021.

[44] Song J, Zheng Y, Zakir Ullah M, Wang J, Jiang Y, Xu C, et al. Multiview multimodal network for breast cancer diagnosis in contrast-enhanced spectral mammography images. Int J Comput Assist Radiol Surg. 2021;16(6):979–88. doi: 10.1007/s11548-021-02391-4 33966155

[45] Sales LF, Pereira A, Vieira T, de Barros Costa E. Multimodal deep neural networks for attribute prediction and applications to e-commerce catalogs enhancement. Multimed Tools Appl. 2021;80(17):25851–73. doi: 10.1007/s11042-021-10885-1

[46] Padi S, Sadjadi S, Manocha D, Sriram S. Multimodal emotion recognition using transfer learning from speaker recognition and BERT-based models. The Speaker and Language Recognition Workshop; 2022.

[47] Busso C, Parbhoo S, Lee CH, Kim S, Narayanan S. IEMOCAP: interactive emotional dyadic motion capture database. In: Proceedings of the International Conference on Affective Computing and Intelligent Interaction (ACII); 2008. p.1-6. doi: 10.1109/ACII.2008.42

[48] Dixit C, Satapathy SM. Deep CNN with late fusion for real time multimodal emotion recognition. Expert Systems with Applications. 2024;240:122579. doi: 10.1016/j.eswa.2023.122579

[49] Bojanowski P, Grave E, Joulin A, Mikolov T. Enriching word vectors with subword information. Trans Assoc Comput Linguist. 2017;5:135–46.

[50] Zadeh A, Chen M, Morency LP. CMU multimodal opinion sentiment and emotion intensity (CMU-MOSEI) dataset [Internet]. CMU Multimodal Communication & Machine Learning Lab; 2018. Available from: https://www.cs.cmu.edu/~emotion/

[51] Zou H, Shen M, Chen C, Hu Y, Rajan D, Chang ES. UniS-MMC: multimodal classification via unimodality-supervised multimodal contrastive learning. arXiv preprint arXiv:2309.06789; 2023.

[52] Villegas DS, Preotiuc-Pietro D, Aletras N. Improving multimodal classification of social media posts by leveraging image-text auxiliary tasks. In: Findings of the Association for Computational Linguistics: EACL; 2024. p.1126-37.

[53] Wang X, Kumar D, Thome N, Cord M, Precioso F. Recipe recognition with large multimodal food dataset. In: IEEE International Conference on Multimedia & Expo Workshops (ICMEW); 2015. p.1–6.

[54] Wang Z, Shan X, Zhang X, Yang J. N24News: a new dataset for multimodal news classification. In: Proceedings of the Language Resources and Evaluation Conference (LREC); 2022. p.6768–75.

[55] DeLucia A, Wu S, Mueller A, Aguirre C, Resnik P, Dredze M. Bernice: a multilingual pre-trained encoder for Twitter. In: Proceedings of the Empirical Methods in Natural Language Processing (EMNLP); 2022. p.6191-6205.

[56] Vempala A, Preotiuc-Pietro D. Categorizing and inferring the relationship between the text and image of Twitter posts. In: Proceedings of the Annual Meeting of the Association for Computational Linguistics (ACL); 2019. p.2830–40.

[57] Niu T, Zhu S, Pang L, El Saddik A. Sentiment analysis on multi-view social data. In: MultiMedia Modeling: 22nd International Conference MultiMedia Modeling (MMM); 2016. p.15-27.

[58] Gomez R, Gibert J, Gomez L, Karatzas D. Exploring hate speech detection in multimodal publications. In: IEEE/CVF Winter Conf Appl Comput Vis (WACV); 2020. p.1470–1478.

[59] Cai Y, Cai H, Wan X. Multimodal sarcasm detection in Twitter with hierarchical fusion model. In: Proceedings of the 57th Annual Meeting of the Association for Computational Linguistics (ACL); 2019. p.2506–2515.

[60] Villegas DS, Goanta C, Aletras N. A multimodal analysis of influencer content on Twitter. arXiv preprint arXiv:2309.03064; 2023.

[61] Aggarwal P. Fashion product images [Internet]. 2022. Available from: https://www.kaggle.com/datasets/paramaggarwal/fashion-product-images-dataset/data

[62] Paszke A, Gross S, Chintala S, Chanan G, Yang E, DeVito Z, et al. Automatic differentiation in PyTorch. In: NIPS Autodiff Workshop; 2017.

[63] Wolf T, Debut L, Sanh V, Chaumond J, Delangue C, Moi A, et al. Transformers: state-of-the-art natural language processing. In: Proceedings of the 2020 Conference on Empirical Methods in Natural Language Processing: System Demonstrations (EMNLP); 2020. p.38-45.

[64] Yang J, Yu M, Zhang W, Meng F, Lu Z, Sun M. Towards making the most of BERT in neural machine translation. In: Proceedings of the AAAI Conference on Artificial Intelligence (AAAI); 2020. p.9378-85.

[65] Kingma DP, Ba J. Adam: a method for stochastic optimization. In: International Conference on Learning Representations (ICLR); 2014.

[66] Loshchilov I, Hutter F. Decoupled weight decay regularization. In: International Conference on Learning Representations (ICLR); 2017.

